# Population pharmacokinetics of liposomal amphotericin B in adults with HIV-associated cryptococcal meningoencephalitis

**DOI:** 10.1093/jac/dkac389

**Published:** 2022-11-22

**Authors:** Katharine E Stott, Melanie Moyo, Ajisa Ahmadu, Cheusisime Kajanga, Ebbie Gondwe, Wezzie Chimang’anga, Madalitso Chasweka, Tshepo B Leeme, Mooketsi Molefi, Awilly Chofle, Gabriella Bidwell, John Changalucha, Jenny Unsworth, Ana Jimenez-Valverde, David S Lawrence, Henry C Mwandumba, David G Lalloo, Thomas S Harrison, Joseph N Jarvis, William Hope, Anne-Grete Märtson

**Affiliations:** Antimicrobial Pharmacodynamics and Therapeutics, Department of Pharmacology, University of Liverpool, UK; Malawi Liverpool Wellcome Programme, Kamuzu University of Health Sciences, Malawi; Malawi Liverpool Wellcome Programme, Kamuzu University of Health Sciences, Malawi; Department of Medicine, Kamuzu University of Health Sciences, Malawi; Malawi Liverpool Wellcome Programme, Kamuzu University of Health Sciences, Malawi; Malawi Liverpool Wellcome Programme, Kamuzu University of Health Sciences, Malawi; Malawi Liverpool Wellcome Programme, Kamuzu University of Health Sciences, Malawi; Malawi Liverpool Wellcome Programme, Kamuzu University of Health Sciences, Malawi; Malawi Liverpool Wellcome Programme, Kamuzu University of Health Sciences, Malawi; Botswana-University of Pennsylvania Partnership, Gaborone, Botswana; University of Botswana, Gaborone, Botswana; National Institute of Medical Research, Mwanza, Tanzania; National Institute of Medical Research, Mwanza, Tanzania; National Institute of Medical Research, Mwanza, Tanzania; Antimicrobial Pharmacodynamics and Therapeutics, Department of Pharmacology, University of Liverpool, UK; Antimicrobial Pharmacodynamics and Therapeutics, Department of Pharmacology, University of Liverpool, UK; Department of Clinical Research, Faculty of Infectious and Tropical Diseases, London School of Hygiene and Tropical Medicine, London, UK; Botswana Harvard AIDS Institute Partnership, Gaborone, Botswana; Malawi Liverpool Wellcome Programme, Kamuzu University of Health Sciences, Malawi; Department of Medicine, Kamuzu University of Health Sciences, Malawi; Department of Clinical Sciences, Liverpool School of Tropical Medicine, Liverpool, UK; Liverpool School of Tropical Medicine, Liverpool, UK; Institute of Infection and Immunity, St George’s University Hospital, London, UK; Medical Research Council Centre for Medical Mycology, University of Exeter, Exeter, UK; Department of Clinical Research, Faculty of Infectious and Tropical Diseases, London School of Hygiene and Tropical Medicine, London, UK; Botswana Harvard AIDS Institute Partnership, Gaborone, Botswana; Antimicrobial Pharmacodynamics and Therapeutics, Department of Pharmacology, University of Liverpool, UK; Antimicrobial Pharmacodynamics and Therapeutics, Department of Pharmacology, University of Liverpool, UK

## Abstract

**Background:**

Single, high-dose liposomal amphotericin B (LAmB; AmBisome, Gilead Sciences) has demonstrated non-inferiority to amphotericin B deoxycholate in combination with other antifungals for averting all-cause mortality from HIV-associated cryptococcal meningitis. There are limited data on the pharmacokinetics (PK) of AmBisome. The aim of this study was to describe population PK of AmBisome and conduct a meta-analysis of the available studies to suggest the optimal dosing for cryptococcal meningoencephalitis.

**Methods:**

Data from a Phase II and Phase III trial of high-dose, short-course AmBisome for cryptococcal meningoencephalitis were combined to develop a population PK model. A search was conducted for trials of AmBisome monotherapy and meta-analysis of clinical outcome data was performed.

**Results:**

A two-compartment model with first-order clearance of drug from the central compartment fitted the data best and enabled the extent of inter-individual variability in PK to be quantified. Mean (SD) population PK parameter estimates were: clearance 0.416 (0.363)  L/h; volume of distribution 4.566 (4.518) L; first-order transfer of drug from central to peripheral compartments 2.222 (3.351)  h^−1^, and from peripheral to central compartment 2.951 (4.070)  h^−1^. Data for the meta-analysis were insufficient to suggest optimal dosing of AmBisome for cryptococcal meningoencephalitis.

**Conclusions:**

This study provides novel insight into the PK of AmBisome at the population level and the variability therein. Our analysis also serves to highlight the paucity of data available on the pharmacodynamics (PD) of AmBisome and underscores the importance of thorough and detailed PK/PD analysis in the development of novel antifungals, by demonstrating the challenges associated with *post hoc* PK/PD analysis.

## Introduction

The recently published AMBIsome Therapy Induction OptimisatioN (AMBITION-cm) trial for HIV-associated cryptococcal meningitis^1^ demonstrated that a single, high dose of liposomal amphotericin B [LAmB (AmBisome, Gilead Sciences); 10 mg/kg] combined with 14 days of flucytosine (100 mg/kg/day) and fluconazole (1200 mg/day) is non-inferior to the previous gold standard treatment regimen of 7 days of amphotericin B deoxycholate (DAmB) (1 mg/kg/day) plus flucytosine (100 mg/kg/day), followed by 7 days of fluconazole (1200 mg/day).^2^ The single-dose regimen has been recommended by the WHO as the preferred induction regimen for the treatment of HIV-associated cryptococcal meningoencephalitis in its recent guidelines.^3^ The administration of AmBisome as a single high-dose IV infusion offers not only an improved safety profile compared with 7 days of DAmB therapy,^1^ but also reduced burden on nursing staff, shortened duration of indwelling IV cannulae, the potential for reduced hospital admission times, less requirement for laboratory monitoring for toxicity, and lower financial costs associated with each of these factors.^3^

AmBisome is a liposomal formulation of amphotericin B comprising 80 nm spherical unilamellar liposomes consisting of a phospholipid bilayer into which amphotericin B compound is incorporated.^4,5^ LAmB is particularly suited to abbreviated therapy for cryptococcal meningoencephalitis because it has a relatively good safety profile that permits higher dosages,^6^ a long terminal elimination phase in tissues^7,8^ and it achieves 4- to 10-fold greater concentrations in brain tissue than other polyene formulations at equivalent dosages.^9^ Despite over 25 years of clinical experience with LAmB for the treatment of a range of invasive fungal infections in adults and children, there are limited data describing the pharmacological properties of the drug and in particular its population pharmacokinetics (PK).^10–13^ There are no population PK models describing LAmB at high doses in adults with cryptococcal meningoencephalitis.

The current PK study was conducted as substudies of the Phase II and Phase III AMBITION-cm trials.^1,14^ By describing the population PK of AmBisome administered at a high dose and in an abbreviated regimen to trial participants, we provide a basis for understanding the action of this drug in a clinically relevant population. The primary aim of this study was to describe population PK of AmBisome. A secondary aim was to conduct a meta-analysis of the available studies of outcome from cryptococcal meningoencephalitis treated with AmBisome, to suggest the optimal dosing for this indication.

## Materials and methods

### Clinical study

Both the Phase II and III study populations were patients with HIV-associated cryptococcal meningoencephalitis. In Phase II of AMBITION-cm, patients received one of the following four treatment regimens: (1) AmBisome 3 mg/kg/day plus fluconazole 1200 mg/day for 14 days; (2) single dose of AmBisome 10 mg/kg on Day 1 only plus fluconazole 1200 mg/day for 14 days; (3) AmBisome 10 mg/kg on Day 1, AmBisome 5 mg/kg on Day 3 plus fluconazole 1200 mg/day from Days 1 to 14; or (4) AmBisome 10 mg/kg on Day 1, AmBisome 5 mg/kg on Days 3 and 7 plus fluconazole 1200 mg/day from Days 1 to 14.^14^ In Phase III of AMBITION-cm, patients were randomized to either a single high dose of AmBisome (10 mg/kg) on Day 1 plus 14 days of both flucytosine 100 mg/kg/day and fluconazole 1200 mg/day—the intervention arm—or 7 days of DAmB 1 mg/kg/day plus flucytosine 100 mg/kg/day, followed by 7 days of fluconazole 1200 mg/day—the control arm.^15^ The present PK study recruited patients from all arms of Phase II, and from the intervention arm of Phase III. Since all patients received combination therapy with other antifungal drugs, we were unable to assess the attributable pharmacodynamic effect of AmBisome using these data.

### Ethics

The PK substudy of the Phase II trial was conducted at Princess Marina Hospital (Gaborone, Botswana) and Bugando Medical Centre and Sekou Toure Hospital (Mwanza, Tanzania). Ethical approval was granted locally by the Botswana Ministry of Health [approval reference: PPME-13/18/1 Vol IX (6)], the National Institute of Medical Research Tanzania, the Research Ethics Committees of the University of Pennsylvania (820127) and the London School of Hygiene & Tropical Medicine (6544-01). The PK substudy of the Phase III trial was conducted at Queen Elizabeth Central Hospital in Blantyre, Malawi. Ethical approval was granted by the Malawi National Health Sciences Research Committee (1907) as well as by the London School of Hygiene & Tropical Medicine (14355). All patients who had capacity to do so provided written, informed consent for participation in the trial and then separately for inclusion in the PK substudy. If patients were incapacitated, consent was obtained from a next of kin with legal responsibility and then patients were re-consented if it became possible according to their clinical status.

### PK sampling

AmBisome was administered in a 2 h IV infusion following pre-hydration with 1 L of 0.9% sodium chloride containing 20 mmol potassium chloride. In the Phase II study, blood samples were collected at the end of the infusion, at 6 h and at 24 h. In the Phase III study, blood samples were collected on Day 1 at 0, 2, 4, 7, 12 and 23 h after the AmBisome infusion was started, and then on Day 7 at 2, 4, 7, 12 and 23 h. A volume of 2 mL of blood was collected into heparinized collection tubes and placed on ice at the bedside. Within 30 min of collection, samples were centrifuged at 1500 g for 10 min at 4°C. Plasma was stored at −80°C until shipment to the University of Liverpool.

### Bioanalysis of PK samples

Amphotericin B concentrations in plasma were quantified by reverse phase UPLC interfaced with a triple quadrupole mass spectrometer using the ACQUITY UPLC system (Waters, Manchester, UK). Amphotericin B was extracted by protein precipitation, using natamycin as internal standard in methanol (2.5 µg/mL) and with 200 µL of natamycin/methanol solution to 50 µL of patient sample. This method was chosen to ensure that total (both liposome-associated and non-liposome-associated) amphotericin B was measured.^16^ Positive pressure was applied to filter out the protein precipitate and collect the supernatant. A volume of 200 µL was added to each well containing supernatant before samples were analysed by UPLC—tandem MS. A 5 µL aliquot was injected onto the reverse phase ACQUITY UPLC HSS T3 column to separate compounds based on their hydrophobicity. Gradient starting conditions were 95% A:5% B, with 0.1% formic acid in water as mobile phase A and 0.1% formic acid in acetonitrile as mobile phase B. Mobile phase B was increased to 99% over 2 min and then reduced to starting conditions for 1 min of equilibration. Flow rate was 0.4 mL/min.

The calibration line for amphotericin B encompassed the concentration range 0.25–50.0 mg/L and was constructed using blank matrix. The lower limit of quantitation was 0.25 mg/L. The coefficient of variation was <9.0% over the concentration range 0.25–50.0 mg/L. The intra- and inter-day variation was <15%.

### Population PK modelling

The PK data were analysed using the non-parametric adaptive grid (NPAG) algorithm of the program Pmetrics version 1.9.7 for R version 4.1.1.^17^ Both two- and three-compartment structural models were explored to fit patient data, with zero-order input into the central compartment and options of both first-order and non-linear (Michaelis–Menten) elimination kinetics from the central compartment. Both mean and median parameter values were examined. The fit of the various models to the data was assessed and compared based on observed-versus-predicted values before and after the Bayesian step, the coefficient of determination of the linear regression of these data, the Akaike information criterion (AIC), the log-likelihood value, the mean weighted error (a measure of bias) and the bias-adjusted, mean weighted squared error (a measure of precision).

Bidirectional stepwise multivariate linear regression was employed to identify any significant associations between clinical covariates and AmBisome PK. Patient age, weight, CD4+ cell count and baseline serum creatinine were investigated as independent predictors of the Bayesian posterior estimates of PK parameters from the baseline model.

### Toxicity

Potential relationships between drug exposure and toxicity were explored using the Phase III study data. Exposure was measured in the first 24 h and over the first week of therapy by calculating AUC_0–24_ and AUC_0–168_ through trapezoidal approximation in Pmetrics using the Bayesian posterior PK predictions from the population model.^17^ The maximum amphotericin B concentration (*C*_max_) during the first week of treatment was also calculated for each patient from the posterior predictions. Toxicity was defined in line with the Division of AIDS Table for Grading the Severity of Adult and Paediatric Adverse Events, version 2.1,^18^ as any of the following grade 3 or 4 adverse events occurring after the start of AmBisome therapy: haemoglobin ≤9.0 g/dL in males or ≤8.5 g/dL in females; creatinine increase to ≥207 μmol/L; potassium decrease to <2.5 mmol/L; ALT increase to ≥180 IU/L. Logistic regression was used to explore the relationship between estimated AUC_0–24,_ AUC_0–168_ and *C*_max_, and the development of these measures of toxicity.

### Meta-analysis of clinical outcome data

The pharmacodynamic (PD) data from patients enrolled in Phase II and Phase III of AMBITION-cm are confounded by the fact that all patients received combination therapy with flucytosine and/or fluconazole.^1,14^ To approximate an association between our PK analysis and patient outcome, we performed a meta-analysis of published clinical outcome data from patients with HIV-associated cryptococcal meningoencephalitis treated with AmBisome monotherapy. We searched PubMed on 4 April 2022 using the terms ‘liposomal amphotericin B’ OR ‘amphotericin B’ OR ‘AmBisome’ AND ‘cryptococcal meningitis’. We filtered the search by article type, selecting only clinical studies and clinical trials. We included only those cohorts that had been administered AmBisome monotherapy and those that were published in English. We extracted information on baseline clinical variables that have consistently been shown to be predictive of clinical outcome—altered mental status and baseline CSF fungal burden.^19,20^ Baseline fungal burden was extrapolated from baseline CSF cryptococcal antigen titre value where necessary, applying a correlation presented by Jarvis *et al*.^19^ Clinical trial outcome data pertaining to CSF sterility and patient mortality were collected. Meta-analysis was conducted on each outcome using a random-effects model to accommodate baseline heterogeneity in the included clinical studies. Dose, baseline CSF fungal burden and baseline mental status were explored as moderator variables to assess the degree to which they accounted for heterogeneity in clinical outcomes. This resulted in a final mixed-effects model of the form: θ*_i_* = β_0_ + β_1_*Z_i_*_1_ + … + β_1_*Z_ij_* + *u_i_*, where θ*_i_* is the corresponding (unknown) true effect of the *i*th study, *Z_ij_* is the value of the *j*th moderator variable for the *i*th study with corresponding model coefficients β, and *u_i_* represents study-specific random effects. In this model, *u_i_* ∼ *N*(0, τ^2^) where *N* indicates that the normal effects are randomly distributed, 0 is the mean of the random effects and τ^2^ signifies the amount of residual heterogeneity unaccounted for by modifiers, estimated by the DerSimonian–Laird estimator.^21^ The null hypothesis *H*_0_:τ^2^ = 0 was tested using Cochran’s Q-test. Model parameters were tested using the Wald-type test statistic.

## Results

### Study population

In total, 56 patients from Phase II of AMBITION-cm were recruited between January 2015 and August 2016, and 31 patients allocated to the intervention arm of Phase III were recruited between November 2018 and October 2019. Demographic and baseline clinical data are displayed in Table 1. The combined PK dataset contained 565 plasma observations from 87 patients, a mean of 6.5 (range 2–12) samples per patient.

**Table 1.** Patient characteristics prior to start of treatment

Characteristic	Values
Sex (male:female), *n*	55:32
Age (years), median (IQR)	37 (32–43)
Weight (kg), median (IQR)	52.0 (46.5–58.7)
Haemoglobin (g/dL), median (IQR)	11.0 (9.7–12.25)
WBC count (10^9^/L), median (IQR)	5.0 (3.5–7.1)
Platelets (10^9^/L), median (IQR)	255.0 (181–328.7)
Creatinine (mmol/L), median (IQR)	64.0 (58.0–87.2)
CD4+ T cell count (cells/mm^3^), median (IQR)	30 (12–60)

### Population PK model

Compared with the two-compartment model, the three-compartment model did not result in improved AIC, −2 log likelihood or measures of imprecision or bias. Similarly, no substantial improvement in these measures of fit was achieved when the first-order clearance model was replaced with a non-linear clearance mechanism. Using the two-compartment model as reference, there was no significant difference in the fit of the three-compartment model or the non-linear clearance model (*P* value for the comparison of the joint distribution of population parameter values between each model >0.05). The third compartment and the non-linear clearance model were both therefore discarded.

The chosen base model took the form:

(1) $$\frac{dX(1)}{dt}=R(1)-\left(\frac{SCL}{V}+KCP\right)\times X(1)+KPC\times X(2)$$

(2) $$\frac{dX(2)}{dt}=KCP\times X(1)-KPC\times X(2)$$

(3) $$Y(1)=\frac{X(1)}{V}$$

Equations 1 and 2 describe the rate of change of the amount of drug (mg) in the central compartment and the peripheral compartment, respectively. R(1) describes the IV infusion of AmBisome into the central compartment. SCL is the first-order clearance of drug from the central compartment (in L/h). The volume of the central compartment (in L) is represented by *V*. KCP and KPC are the first order intercompartmental rate constants (in h^−1^). The model output (concentration of amphotericin B in the central compartment) is described by Equation 3.

**Figure 1.**
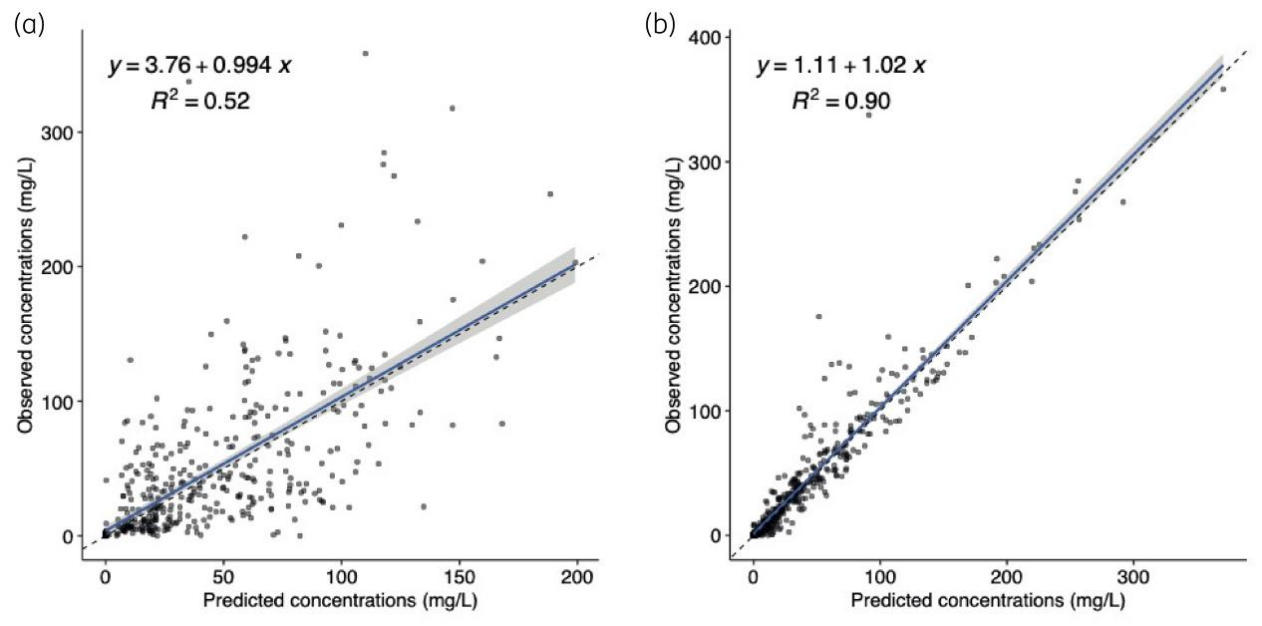
Goodness-of-fit plots of observed versus predicted AmBisome concentrations (mg/L) for the chosen population PK model after the Bayesian step. The shaded area is the 95% CI around the regression line. (a) Fit of the final model to the plasma data from the population. (b) Fit of the Bayesian posterior PK estimates for individual patients from the final model to the observed data. This figure appears in colour in the online version of *JAC* and in black and white in the print version of *JAC*.

Multivariate linear regression of clinical covariates did not reveal any significant associations between the Bayesian posterior estimates of clearance and volume versus age, sex, weight, renal function (baseline creatinine) or CD4+ cell count. The baseline two-compartment PK model was therefore not modified. Additive and proportional errors were tested in the model and additive error was selected for the final model. The final model population fit resulted in *r^2^* of 0.52 and individual fit in *r^2^* of 0.90 in a linear regression of observed-versus-predicted concentrations of DAmB (Figure 1). Population PK parameter estimates from the final model are displayed in Table 2.

**Table 2.** Population parameter estimates from the final two-compartment PK model

Parameter	Mean	Median	SD	Shrinkage (%)
Clearance (L/h)	0.416	0.345	0.363	9.539
Volume (L)	4.566	3.698	4.518	5.930
KCP (h^−1^)	2.222	0.218	3.351	10.769
KPC (h^−1^)	2.951	0.484	4.070	17.928

KCP, first-order rate constant for the AmBisome distribution from the central to the peripheral compartment; KPC, first-order rate constant for the AmBisome distribution from the peripheral to the central compartment.

### Measures of toxicity

Toxicity was analysed for the Phase III study only (*n* = 31). Anaemia was common in the clinical cohort and was present at baseline in 16% of patients (*n* = 5). In addition, 38% of patients (*n* = 12) developed grade 3 or 4 anaemia during treatment. There was no evidence of raised creatinine, hypokalaemia or raised ALT before the start of treatment. However, 12% of patients (*n* = 4) had a grade 3 or 4 rise in creatinine, 3% of patients (*n* = 1) developed grade 3 or 4 hypokalaemia and 3% of patients (*n* = 1) had a grade 3 or 4 rise in ALT during treatment. There were no significant correlations between AmBisome exposure (quantified by AUC_0–24_ and AUC_0–168_) or amphotericin B *C*_max_, and any of these measures of toxicity (Table 3).

**Table 3.** ORs of developing grade 3 or 4 laboratory-defined adverse events during AmBisome-containing induction therapy

Event	OR of event occurring with a unit increase in AUC_0–24_, estimate (*P* value for estimate)	OR of event occurring with a unit increase in AUC_0–168_
Anaemia	1.0 (0.47)	1.0 (0.29)
Creatinine increase	1.0 (0.43)	1.0 (0.51)
Hypokalaemia	1.0 (0.89)	1.0 (0.70)
Elevated ALT	1.0 (0.82)	1.0 (0.85)

Anaemia was defined as haemoglobin ≤9.0 g/dL in males or ≤8.5 g/dL in females; creatinine increase was defined as ≥207 μmol/L; hypokalaemia was defined as potassium decrease to <2.5 mmol/L; elevated ALT was defined as an increase to ≥ 180 IU/L.

### Meta analysis of clinical outcome data

Just three studies, detailing four different dosing cohorts of AmBisome monotherapy for cryptococcal meningoencephalitis were identified.^6,22,23^ Across the four cohorts, 105 patients across two cohorts received 3 mg/kg/day, 15 patients received 4 mg/kg/day and 94 patients received 6 mg/kg/day. Treatment duration ranged from 11 to 42 days. Patient ages reported from the cohorts were reasonably homogeneous, ranging from a mean of 33 to 40 years. All patients were HIV positive. Baseline characteristics and reported clinical outcomes are summarized in Table 4.

**Table 4.** Patient characteristics and clinical outcome data from trials of AmBisome monotherapy for cryptococcal meningoencephalitis

Study	LAmB dose (mg/kg/day)	Treatment duration, days	Country	Number of patients	Mean age (range), years	Baseline mental status, mean Karnofsky score	Baseline CD4, mean (range), cells/μL	Baseline CSF fungal burden, median, cfu/mL	Patients with sterile CSF at 2 weeks, %	Patients with sterile CSF at 10 weeks, %	10 week mortality, %
Hamill, 2010	3	11–21	USA, Canada	86	38.7 (22–61)	64	49 (2–428)	5	58.3	60	14
Coker, 1993	3	42	UK, Germany, France, Portugal	19	33 (21–47)	NR	35 (1–174)	6.1	67	NR	15.7
Leenders, 1997	4	21	Australia, the Netherlands	15	40 (29–55)	70^a^	35^a^ (10–70)	4.8	66.7	100	6.7
Hamill, 2010	6	11–21	USA, Canada	94	40.1 (21–68)	66	51 (2–598)	5.5	48	70.7	9.6

NR, not reported.

a Median value.

The meta-analysis was limited by a paucity of available data and a narrow range of dosages in the available studies (Table 4). The final model was unable to elicit significant inter-study heterogeneity in the estimated proportion of patients with sterile CSF at 2 weeks prior to the inclusion of modifying variables (*P* value for residual heterogeneity, 0.29). The inclusion of dose and baseline fungal burden as modifying variables had the effect of reducing τ^2^ from 0.0023 to 0.0003, indicating a trend towards reduction in total inter-study heterogeneity; however, this did not reach statistical significance (*P* value for moderators, 0.28). Similarly, the test for residual heterogeneity in mortality outcomes at 10 weeks, prior to the inclusion of modifying variables, was not significant (*P* value, 0.65). Total inter-study heterogeneity in 10 week mortality outcomes was undetectable (τ^2^ = 0) prior to the inclusion of modifying variables; these were therefore not tested. Data for the meta-analysis were insufficient to suggest optimal dosing of AmBisome for cryptococcal meningoencephalitis.

## Discussion

This study is the first to assess and model the population PK of LAmB administered at high doses in abbreviated regimens to adult patients with cryptococcal meningoencephalitis. A two-compartment model with first-order clearance of drug from the central compartment fit the data best and enabled the extent of inter-individual variability in PK to be quantified at the population level. PK models describing saturable clearance mechanisms did not improve model fit over models describing first-order clearance of amphotericin B. This finding contrasts with other reports, which suggest that non-linear clearance mechanisms may be activated with high doses of AmBisome (7.5–15 mg/kg/day).^24^ However, the present analysis is consistent with another study that modelled data from adult patients administered a dose of 10 mg/kg and found no evidence of non-linear PK at this dose.^11^ It may be that these alternative clearance mechanisms are only activated at doses higher than 10 mg/kg, or with high doses that are administered in daily, rather than abbreviated, regimens. In addition, since patients in the present study received a uniform weight-based dosage of AmBisome and there was modest variation in body weight among the cohort, we were limited in our ability to detect PK non-linearity.

Measured AmBisome concentrations were highly variable and this was reflected in the population parameter estimates from the final model. However, exploration of clinical covariates (e.g. age, sex, baseline creatinine and weight) did not reveal the source of this variability. The complex logistical nature of these studies, which were conducted in busy hospitals in resource-limited clinical settings, may account for some variation in drug administration and PK sampling times, though all reasonable efforts were made to mitigate this. The patient cohort was critically unwell, which in itself is a recognized source of PK variability.^25^ A degree of variability may be accounted for by process noise inherent to the laboratory drug assay. However, a substantial portion of the PK variability observed in this study likely represents true PK variability that is characteristic of AmBisome itself, as has been reported by others.^11,13^ Indeed, we are not the first to report the absence of a relationship between weight and AmBisome PK^12^ and it has been suggested that any such signal may be obscured by the inherent PK variability of the drug.^11^ Specific pharmacometric evaluation of the requirement for weight-based dosing of AmBisome is warranted since the evidence for current practice is limited, and a single recommended dose for all patients would further simplify administration.

In the Phase III dataset, we found no relationship between AmBisome exposure and the frequency of adverse events. The fact that AmBisome was well tolerated in our cohort is unsurprising, since 10 mg/kg of LAmB is routinely administered for visceral leishmaniasis and dosages as high as 15 mg/kg/day are well tolerated in adult patients.^24,26^ The low propensity of LAmB to cause nephrotoxicity, compared with DAmB, may be related to the lipoprotein binding affinities of each formulation; LAmB preferentially binds HDL, which is taken up by the reticuloendothelial system (RES).^27,28^ Levels of amphotericin B in the kidney are therefore lower than those in the RES after LAmB administration.^28^ In contrast, DAmB has an affinity for LDL, receptors for which are highly expressed on glomerular endothelium.^29,30^ Furthermore, the liposome vehicle of amphotericin B formulated as LAmB may enhance the drug’s selectivity for fungal cell membranes over mammalian cell membranes, through the interaction of the liposome with fungal phospholipases.^24,31,32^

Since the clinical PD data were confounded by the co-administration of additional antifungal agents in our cohort, we conducted a meta-analysis of previous studies that recorded clinical outcomes from treating patients with AmBisome monotherapy for cryptococcal meningoencephalitis. Such studies are limited, and we did not have sufficient statistical power to detect an association between the modifying variables (dose, baseline fungal burden and baseline mental status) and clinical outcome in terms of CSF sterility or mortality. Since combination therapy has become the gold standard for cryptococcal meningoencephalitis, clinical outcome or PD data gathered from patients treated with AmBisome monotherapy is unlikely to be forthcoming. Further insight into the PD of AmBisome may be possible through pragmatic preclinical studies.

The pharmacological mechanisms that explain why AmBisome should be effective in single-dose or abbreviated regimens remain somewhat elusive. The simplicity with which our population model was able to capture the plasma PK of AmBisome belies the drug’s complex molecular pharmacology, which is not fully understood. The rapid, dose-dependent fungicidal activity of LAmB has been established in preclinical studies of invasive candidiasis,^33^ mucormycosis,^34^ histoplasmosis^35^ and blastomycosis^35^ in addition to cryptococcosis.^36^ From the present PK model, it is clear that plasma concentrations fall to negligible levels 48–72 h after a single dose of 10 mg/kg of AmBisome. From preclinical models and from the AMBITION-cm trial we know that despite the fall in plasma levels, the drug appears to exert ongoing anti-cryptococcal activity that is non-inferior to prolonged amphotericin B regimens.^8,14,15^ This may imply that there are persistent fungicidal drug concentrations at the effect site, that is in the meninges and/or cerebral tissue. Certainly AmBisome has a long terminal half-life in both plasma and cerebrum—approximately 152 hours in one study^37^—however, one would nevertheless expect that if concentration–time profiles in the CNS and in plasma were synchronous, drug exposure in the CNS would fall to subtherapeutic levels over too short a time for antifungal effect to be adequate. It has been suggested that there is an exhaustive number of CNS binding sites for LAmB, from which the drug does not readily disengage. Once saturated, these sites form a reservoir of drug that has ongoing antifungal activity.^8^ Indeed, studies comparing daily administration of different dosages of AmBisome for cryptococcal meningoencephalitis^6^ and other disease states^38–40^ found no additional antifungal activity at higher dosages. Alternatively, or perhaps in addition to persistence of drug in the CNS, it is possible that concentrations of amphotericin B achieved in meninges and/or cerebral tissue are considerably higher than those in plasma, though murine data do not support this theory.^8^ Of note, given the uncertainties surrounding the mechanism of prolonged efficacy of AmBisome and the knowledge gaps pertaining to its PD effect, extrapolation of our findings to other formulations of LAmB, or indeed to infections with a primary tissue focus outside the CNS, such as pulmonary aspergillosis, would not be appropriate.

In summary, this study describes the first population PK model of high-dose abbreviated AmBisome regimens in patients with HIV-associated cryptococcal meningoencephalitis. We provide novel insight into the PK of AmBisome at the population level and the variability therein, in a relevant clinical population. This work will be relevant to efforts to further improve the treatment of cryptococcal meningoencephalitis, for example through exploration of combination regimens (including with novel antifungal agents^41^) and in specific populations, including non-HIV immunosuppressed populations. Our analysis also serves to highlight the paucity of data available on the PD of AmBisome and underscores the importance of thorough and detailed PK/PD analysis in the development of novel antifungals, by demonstrating the challenges associated with *post hoc* PK/PD analysis.

## Transparency declarations

T.S.H. and J.N.J. were recipients of the investigator award (to institution) for the Phase II study, and have received speaker fees from Gilead Sciences. W.H. holds or has recently held research grants with F2G, Astellas Pharma, Spero Therapeutics, Antabio, Allecra, Bugworks and NAEJA-RGM. He holds awards from the Medical Research Council, National Institutes of Health Research, FDA and the European Commission. W.H. has received personal fees in his capacity as a consultant for F2G, Amplyx, Ausperix, Spero Therapeutics, Venatorx, Pfizer and BLC/TAZ. All other authors: no transparency declarations to declare.
